# ***The reference genome of*** Macropodus opercularis (***the paradise fish***)

**DOI:** 10.1038/s41597-024-03277-1

**Published:** 2024-05-25

**Authors:** Erika Fodor, Javan Okendo, Nóra Szabó, Kata Szabó, Dávid Czimer, Anita Tarján-Rácz, Ildikó Szeverényi, Bi Wei Low, Jia Huan Liew, Sergey Koren, Arang Rhie, László Orbán, Ádám Miklósi, Máté Varga, Shawn M. Burgess

**Affiliations:** 1 Department of Genetics, ELTE Eötvös Loránd University, Budapest, Hungary; 2 Translational and Functional Genomics Branch, National Human Genome Research Institute, Bethesda, MD USA; 3 Frontline Fish Genomics Research Group, Department of Applied Fish Biology, Institute of Aquaculture and Environmental Safety, Hungarian University of Agriculture and Life Sciences, Georgikon Campus, Keszthely, Hungary; 4 Science Unit, Lingnan University, Hong Kong, China; 5 Computational and Statistical Genomics Branch, National Human Genome Research Institute, Bethesda, MD USA; 6 Department of Ethology, ELTE Eötvös Loránd University, Budapest, Hungary

**Keywords:** Genome, Structural variation

## Abstract

Amongst fishes, zebrafish (*Danio rerio*) has gained popularity as a model system over most other species and while their value as a model is well documented, their usefulness is limited in certain fields of research such as behavior. By embracing other, less conventional experimental organisms, opportunities arise to gain broader insights into evolution and development, as well as studying behavioral aspects not available in current popular model systems. The anabantoid paradise fish (*Macropodus opercularis*), an “air-breather” species has a highly complex behavioral repertoire and has been the subject of many ethological investigations but lacks genomic resources. Here we report the reference genome assembly of *M. opercularis* using long-read sequences at 150-fold coverage. The final assembly consisted of 483,077,705 base pairs (~483 Mb) on 152 contigs. Within the assembled genome we identified and annotated 20,157 protein coding genes and assigned ~90% of them to orthogroups.

## Background & Summary

During the 20^th^ century experimental biology gained increased influence over descriptive biology and concomitantly most research efforts began to narrow into a small number of “model” species. These organisms were not only selected because they were considered to be representative models for the examined phenomena but were also easy and cheap to maintain in laboratory conditions^1,2^. Working with these convenient experimental models had several advantages and made a rapid accumulation of knowledge possible. It enabled scientists to compare and build on each other’s findings efficiently as well as to share valuable data and resources that accelerated discovery. As a result of this, a handful of model species have dominated the field of biomedical studies.

Despite their broad success, these models also brought limitations. As Bolker pointed out: “The extraordinary resolving power of core models comes with the same trade-off as a high-magnification lens: a much-reduced field of view”^3^. In the case of zebrafish research this trade-off has been perhaps most apparent for behavioral studies. Zebrafish are an inherently social (shoaling) species, but most behavioral studies use them in solitary settings, which arguably is a non-natural environment for them. Therefore, the use of other teleost species with more solitary behavioral profiles is warranted for studies of individual behaviors.

Paradise fish (*Macropodus opercularis* Linnaeus, 1758) are a relatively small (8–11 cm long) freshwater fish native to East Asia, Southern China, Northern Vietnam, and Laos where they are commonly found in shallow waters with dense vegetation and reduced dissolved oxygen^4^. Similar to all other members of the suborder *Anabantoidae*, they are characterized by the capacity to take up oxygen directly from the air through a highly vascularized structure covered with respiratory epithelium, the labyrinth organ (LO)^5^. The ability to “air-breathe” allows anabantoids to inhabit swamps and small ponds with low levels of dissolved oxygen that would be impossible for other fish species, therefore the LO can be considered an adaptation to hypoxic conditions^6^. The evolution of the LO has also improved hearing in some species^7,8^, and may have led to the emergence of novel and elaborate mating behaviors, including courtship, territorial display, and parental care^6,9^.

Another interesting behavior that these fish possess is they build egg “nests” by blowing bubbles on the surface of the water^6,10^. These types of intricate and complex behaviors fish made them an important ethological model during the 1970–80 s, which resulted in a detailed ethogram of the species^11,12^.

We propose that with recently developed husbandry protocols^13^ and the advent of novel molecular techniques for genome editing and transgenesis, paradise fish could become an important complementary model species for neurogenetic studies^14^. Furthermore, several genomes are now available for the Siamese fighting fish (*Betta splendens*), a closely related species to the paradise fish^15–17^, so a good quality genome sequence of paradise fish would enable comparative ecological and evolutionary (eco-evo) studies.

While the mitochondrial genome was already available for this species^18^ a full genome sequence was lacking. Here, we provide a brief description and characterization of a high quality, *de novo* paradise fish reference genome and transcriptome assembly.

## Methods

### Animals and husbandry conditions

The paradise fish used to establish our colony and the source of the transcriptome samples were purchased from a local pet store (Trioker Ltd., Érd, Hungary). Adult paradise fish were kept in aerated glass aquariums in the animal facility of the Institute of Biology at ELTE Eötvös Loránd University. Husbandry conditions were specified previously^13^. Embryos were raised at 28.5 °C and staged as described before^19^. All experimental procedures were approved by the Hungarian National Food Chain Safety Office (Permit Number: PE/EA/406—7/2020). Animal experiments in Hungarian academic research centres are regulated by decree no. 40/2013 (14.II.) issued by the Hungarian Government, which was drafted based on Directive 2010/63/EU on the protection of animals used for scientific purposes. The research on paradise fish in Dr. Varga’s laboratory was made possible by permit no. PE/EA/406-7/2020, issued by the Pest County Government Office on the basis of the above-mentioned government regulation. Wild-caught adult paradise fish were captured in the areas surrounding Hong Kong and the specimens were handled in accordance to protocols outlines in the Research Ethics Approval Application via Lingnan University (Reference number: EC051/2021). Permission to collect wild specimens were also granted in a permit obtained from the AFCD (Agriculture, Fisheries, and Conservation Department). The permit number is “AF GR CON 11/17 Pt. 7”.

### Sample collection, library preparation and sequencing

RNA samples were collected from a mix of embryonic stages (stage 9 – 5 days post fertilization), from caudal tail blastema taken at 3- and 5-days post amputation, from the kidney, heart, brain, ovaries of an adult female, and the brain and testis of an adult male paradise fish, respectively. Total RNA was isolated using TRIzol (Invitrogen, 15596026), following the manufacturer’s protocol. Samples were purified twice with ethanol and eluted in water. Quality and integrity of the samples was tested on an agarose gel, by Nanodrop, and using an Agilent 2100. Ribosomal RNA (rRNA) was removed using the Illumina Ribo-Zero kit and paired-end (PE) libraries were prepared using standard Illumina protocols. Samples were processed on an Illumina NovaSeq PE150 platform, and a total of 218,715,409 PE reads (2x 150 bp) were sequenced, resulting in ~65 Gbp of raw transcriptomic data.

Genomic DNA samples were isolated from the tail fin of the parental F_0_ male and female paradise fish using the Qiagen DNeasy Blood and Tissue Kit (cat no: 69504). Samples were eluted in TE and sent for library preparation and sequencing. Sample quality-checks were performed using standard agarose gel electrophoresis and with a Qubit 2.0 instrument. For Illumina short-read sequencing a size-selected 150 bp insert DNA library was prepared and processed on the Illumina NovaSeq. 5000 platform. Approximately 100 million PE reads (2 × 150 bp) were sequenced for each parent, resulting in approximately 60X coverage for each genome. For PacBio HiFi long-read single molecule real-time (SMRT) sequencing libraries, genomic DNA was prepared using whole tissue from the 6 month old F_1_ offspring and the Circulomics Nanobind tissue kit. Sequence libraries were prepared using the PacBio SMRTbell Template Preparation Kit and HiFi sequenced on a Sequel II platform. A total of 4,885,238 reads (average length: 15.5 kbp) resulted in ~73 Gbp of raw genomic sequence data.

### Genome assembly

All software versions used are listed in Supplementary Table S4. The raw data pre-processing was conducted by doing quality control, adapter trimming, and filtration of the low-quality reads using trim galore wrapper around FASTQC and Cutadapt^20^. The genome assembly was generated with the hifiasm genome assembler^21^ using the High-Performance Computing facility at the National Institute of Health. For the assembly, 32 cores processing units and 512 Gb of memory was used. The lower and the upper bound binned K-mers was set to 25 and 75, respectively. The estimated haploid genome size used for inferring reads depth was set to 0.5Gbp. The rest of the hifiasm default settings were used to assemble the homozygous genome with the build-in duplication purging parameter set to -l1. The primary assembly Graphical Fragment Assembly (GFA) file was converted to FASTA file format using the awk command.

### Genome annotation

The Trinity assembler^22^ was used to create a set of RNA transcripts from the bulk RNA-seq data. To aid in gene prediction, we downloaded the reviewed Swissprot/Uniprot vertebrate proteins (Download date 12/01/2022; entries 97,804 proteins) for homology comparisons in annotation pipelines. Gene prediction was done using the AUGUSTUS^23^ and GeneMark-ES^24^ softwares as part of the BRAKER pipeline^25^ to train the AUGUSTUS parameters. Final annotation using the assembled transcripts and the vertebrate proteins database was done using the MAKER pipeline^26^ with the EvidenceModeller^27^ tool switched-on to improve gene structure annotation.

### Intron size and OrthoFinder analysis

Sources for the reference data used to create Figs. 2, 3: refs. ^28–34^. We performed OrthoFinder analysis^35,36^ with default parameters, using predicted peptides (including all alternative splice versions) of the zebrafish genome assembly GRCz11, medaka genome assembly ASM223467v1 and *B. splendens* genome assembly fBetSpl5.3. Sequences were downloaded from the ENSEMBL and NIH/NCBI Assembly homepages, respectively.

### Variant calling

The Illumina short read files (accession number ERR3332352) were downloaded from the Vertebrate Genome Project (VGP) database (https://vertebrategenomesproject.org/). Illumina short read sequencing was also performed on genomic DNA obtained from the tails of 3 wild-caught fish from the Hong Kong region. Trim galore version 0.6.10 (https://github.com/FelixKrueger/TrimGalore), a wrapper around cutadapt and fastqc was then used to trim the illumina adapter sequences and to discard reads less than 25 bps. DRAGMAP version 1.3.0 (https://github.com/Illumina/DRAGMAP) was used to map the reads to the reference genome. The resultant sequence alignment map (SAM) file was then converted to binary alignment map (BAM), sorted, and indexed using samtools. The Picard was then used to add the read groups information in BAM file. The genome analysis tool kit (GATK) was then used in calling the variants by turning on the dragen mode. The bamtools stats and the plot-vcfstats was used in the downstream analysis and visualization of the genomic variants in the variant call file (VCF).

## Data Records

The assembly and all DNA and RNA raw reads have been deposited in the NCBI under the BioProject study accession PRJNA824432. Within that project there is the GenBank assembly macOpe2 (GCA_030770545.1), 9 RNA-seq raw sequence data files (SRX20729884, SRX15898419, SRX15898418, SRX15898417, SRX15898416, SRX15898415, SRX15898414, SRX15898413, SRX15898412), one PacBio HiFi genomic raw sequence data file (SRX15948463), one Illumina PE short read genomic raw sequence data file for the assembly (SRX15948462) and Illumina short read genomic raw sequence data files for the 3 wild-caught samples (SAMN39260618, SAMN39260619, SAMN39260620)^37,38^. The variant data for this study have been deposited in the European Variation Archive (EVA) at EMBL-EBI under accession number PRJEB74481^39^.

## Technical Validation

### Assembly quality and completeness

**Fig. 1:**
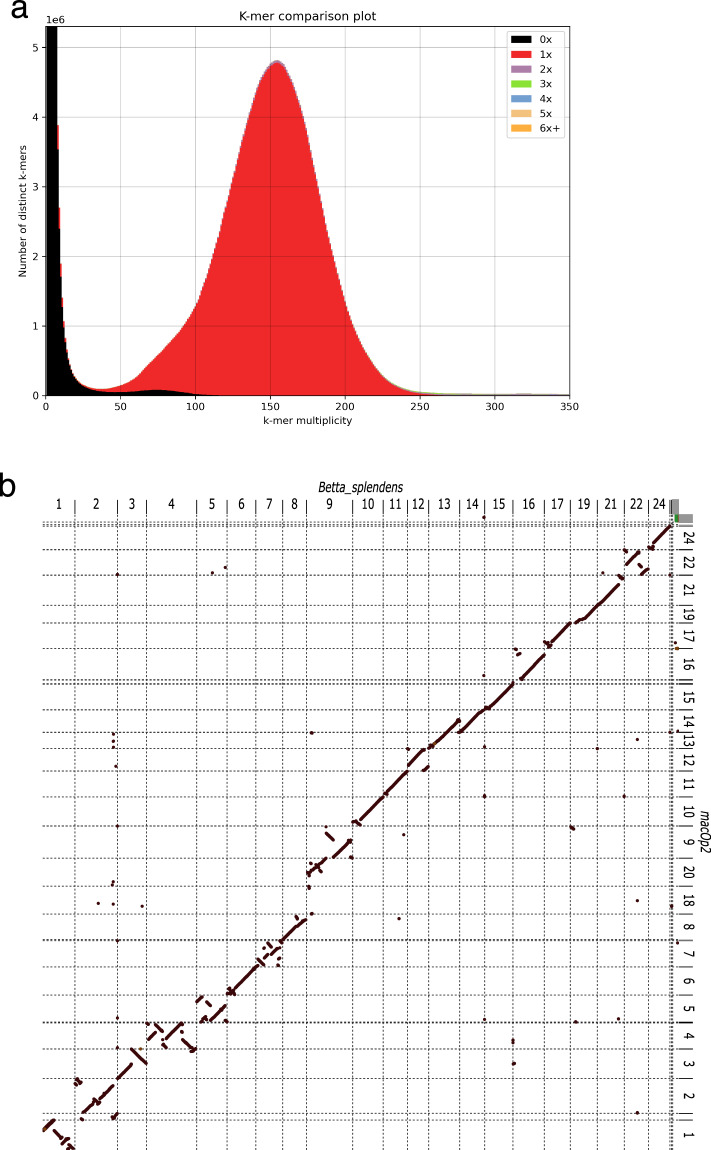
Basic genome assessment. (**a**) K-mer comparison plot showing copy number of the k-mers as a stacked histogram colored by the copy numbers found in the paradise fish draft genome assembly. The y-axis represents the number of distinct k-mers, and the x-axis shows the k-mer multiplicity (coverage). Most k-mers are represented once (red peak) indicating high quality for the genomic assembly. (**b**) Whole genome alignment of the *B. splendens* genome assembly to the *M. opercularis* assembly. *B. splendens* is on the X-axis and *M. opercularis* on the Y-axis. *B. splendens* chromosomes 9 appears to map to two separate *M. opercularis* chromosomes and *M. opercularis* chromosome 18 does not appear to have a clear homologous chromosome in *B. splendens*. Chromosome numbering is based on *B. splendens* alignment to medaka chromosomes^14,20^.

We generated the *de novo* reference genome sequence for this species using 150X coverage of PacBio SMRT HiFi long-read sequencing and the hifiasm genome assembly pipeline^21^. The final assembly consisted of 483,077,705 base pairs (bp) on 152 contigs (Supplementary Table S1). The assembled genome demonstrated a very high contiguity with an N50 of 19.2 megabases (Mb) in 12 contigs. The largest contig was 24,022,457 bp and the shortest contig was 14,205 bp. More than 98% of the canonical k-mers were 1x copy number indicating that our genome assembly is of very good quality (Fig. 1a). The paradise fish genome repeat content is estimated to be ~10.4%. The “trio binning”^40^ mode of Hifiasm was attempted using single nucleotide variant (SNV) data collected from short read sequencing of the F_0_ parents, however the heterozygosity rate from the lab raised fish was very low at ~0.07% making it impossible to efficiently separate maternal and paternal haplotypes. The resulting assembled reference genome is therefore a pseudohaplotype. The sequence of the mitochondrial genome (mtDNA) was essentially identical to the previously published mtDNA sequence for this species (16,495/16,496 identities)^18^. We followed the *B. splendens* example and numbered the *M. operculis* chromosomes based on their similarity to medaka chromosomes resulting in the chromosomes being numbered 1–19, and 21–24. We performed a whole genome alignment to a recent *Betta splendens* assembly^34^ 21 chromosomes had a 1 to 1 relationship with *B. splendens* chromosome 9 aligning to two separate paradise fish contigs (Fig. 1b) and *M. opercularis* chromosome 18 having no significant homology to a *B. splendens* chromosome. This is explained by the number of chromosomes for each species with *B. splendens* having 21 and *M. opercularis* reportedly having 23 chromosomes^41^. We have not determined whether the *B. splendens* chromosomes fused or if the *M. opercularis* chromosomes split.

**Fig. 2:**
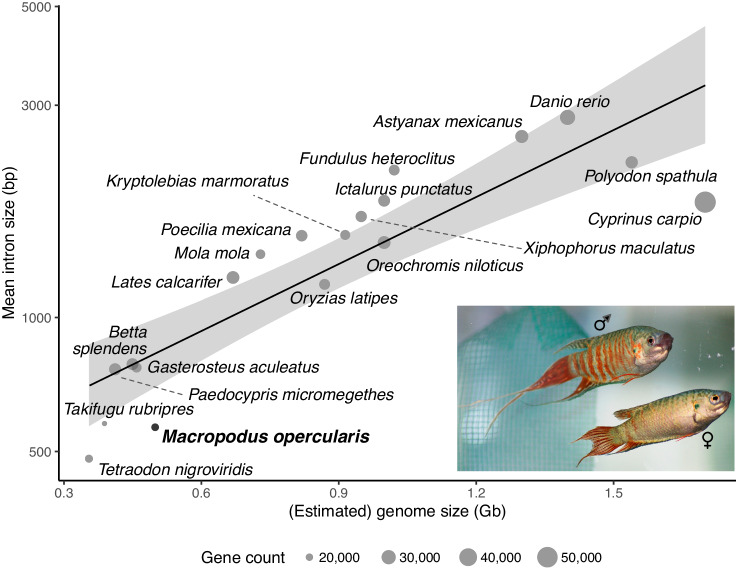
Distribution of intron sizes for various species of fish. *M. opercularis* is at the low end of the spectrum for fish genome size, similar to puffer fish species. The line denotes the linear regression line fitted over the data. Grey areas denote the 0.95 confidence interval of the fit. The diameter of the circles correlates to the approximate gene count (20 to 50 thousand).

The genome is relatively compressed in size and has relatively small introns (mean paradise fish intron length = 566 bp, whereas mean average teleost intron size = 1,214^28^) (Fig. 2) and shorter intergenic regions. The N90 for our assembly consists of 23 contigs suggesting that most chromosomes are primarily represented by a single contig from the *de novo* assembly, even without any scaffolding performed. Searching the contigs with zebrafish telomeric sequences revealed “telomere-to-telomere” assemblies, i.e. contigs that had telomeric sequences at both ends in the correct orientation, for contigs ptg000004l, ptg000010l, ptg000024l, ptg000026l, ptg000028l, and ptg000030l representing chromosomes 3, 8, 9, 15, 17 and 21, respectively (Supplementary Table S3). These contigs have vertebrate telomeres at both ends while the remaining contigs have one or no stretches of telomeric sequence at the end of the contig.

Benchmarking Universal Single-Copy Orthologs (BUSCO) was used to evaluate the completeness of our reference genome assembly with the Actinopterygii_odb10 dataset^42,43^. The result showed that 98.5% of the sequence in the reference dataset had a complete ortholog in our genome including 97.3% complete and single-copy genes and 1.2% complete and duplicate genes. Additionally, 1.2% of the genes were reported as fragmented and 0.3% of the genes were completely missing.

### Paradise fish genome assembly repeat content characterization

Using RepeatMasker^44^, we analysed and characterized the repeat content in our reference genome assembly. By using a custom-built repeat prediction library, we identified 32,955,420 bp (6.78%) in retroelements and 11,076,209 bp (2.8%) in DNA transposons (Supplementary Table S2). The retroelements were further categorised into repeat families which were made up of short interspersed nuclear elements (SINEs), long interspersed nuclear elements (LINEs), or long terminal repeats (LTR) (Supplementary Table S3). The LINEs were the most abundant repetitive sequence in the retroelement family at 3.38% (16,447,763 bp) followed by LTRs, 3.19% (15,490,642 bp), and SINEs occurred at a lowest frequency (0.21%) (Supplementary Table S2). In the LINEs sub-family, we identified L2/CR1/Rex as the most abundant repetitive sequence (2.15%) followed closely with the retroviral (1.73%) LTR sub-family (Supplementary Table S2). The proportion of the DNA transposons was estimated to be 11,076,209 bp (2.28%). Overall, the proportion of retroelements (6.78%) was much higher in the genome compared to that of DNA transposons (2.28%).

The Vertebrate Genome Project (VGP)^45^ had performed short read sequencing on a single paradise fish purchased from a German pet shop (NCBI accession: PRJEB19273), and we captured 9 “wild” samples from the New Territories in Hong Kong. We performed short read sequencing to ≥20X coverage for 3 of the wild-caught fish and used the data in combination with the VGP effort to establish the SNP rates within the paradise fish populations. We identified 5,867,521 variants having a quality score of greater or equal to 30 (Table 1) across 4 individual fish. The transition/transversion rate was 1.41. Our analysis identified a total of 663,781 insertions or deletions ranging from 1 to 60 bps. The rate of SNPs and the indels were 0.5% and 0.1%, respectively.

**Table 1:** Paradise fish variants call summary statistics from four fish compared to the reference assembly.

SNV*		Indels***
n	ts/tv**	n
5,867,521 (0.3%)	1.35	633,781 (0.03%)

*SNV - single nucleotide variants.

**ts/tv - transitions to transversions ratio.

***Indels - insertions/deletions.

### Transcriptome assembly and quality assessment

The Trinity transcriptome assembler was used to assemble the Illumina short reads from the RNA-sequencing data^22^ into predicted transcripts. The transcriptome assembly consisted of 366,029 contigs in 20,157 loci. The integrity of the transcriptome assembly was evaluated by mapping the Illumina short reads back to the assembled transcriptome using bowtie2^46^; a 98.4% overall alignment rate was achieved. The BUSCO analysis confirmed 99.6% completeness with 8.2% single copy orthologs and 91.4% duplicated genes (*i.e*. multiple isoforms). A total of 0.4% of the genes were fragmented and 0.0% were missing completely.

#### Genome annotation

**Fig. 3:**
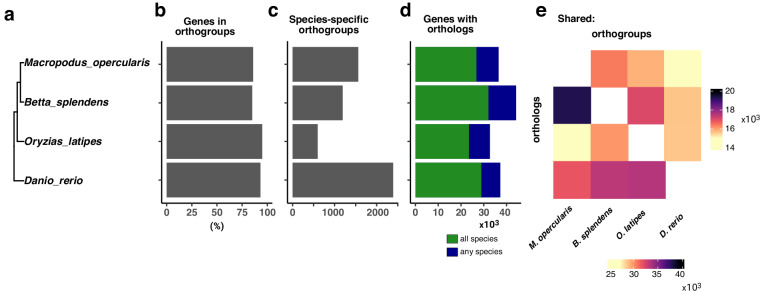
Comparison of predicted proteins across 4 species shows the close relationships between *M. opercularis* and *B. splendens*. (**a**) Evolutionary relationship between paradise fish, Siamese fighting fish, zebrafish, and Japanese medaka. (**b**) The number of genes per species that could be placed in an orthogroup (the set of genes descended from a single gene in the last common ancestor of all the species). This value is close to 90% for all four species. (**c**) The number of orthogroups that are specific to each species. (**d**) The total number of transcripts with orthologs in at least one other species. (**e**) Heat map of the orthogroups for each species pair (top) and orthologs between each species (bottom). The relative evolutionary distances between the different species are consistent with the overall levels of gene conservation.

We analyzed the predicted genes using OrthoFinder^35^ compared to the *Betta splendens*^17^, medaka^47^ and zebrafish^48^ genomes (Fig. 3). Our analysis shows that 89.6% of the predicted genes (18,057/20,157) of paradise fish could be assigned to orthogroups (Fig. 3b), of which only a very low percentage – 2.5% (511/20,517) – were present in species-specific orthogroups (Fig. 3c). A vast majority of the annotated genes (17,546/20,517) had orthologs in at least one of the analyzed species, with 70% (14,067/20,517) having orthologs in all the other species (Fig. 3d). The ratio of shared orthogroups also supports the expected phylogeny (Fig. 3e).

### Supplementary information

Supplementary information

## Data Availability

No custom code was generated for this project. All software with parameters are listed in Supplementary information.
