# Supplementary material for: The reference genome of Macropodus opercularis (the paradise fish)
Source: Sci Data. 2024 May 25;11:540. doi: 10.1038/s41597-024-03277-1 (PMC11127978; doi:10.1038/s41597-024-03277-1)
Supplement: Supplementary file 1 — Supplementary information [file 41597_2024_3277_MOESM1_ESM.pdf]

Table S1: Summary of the paradise fish genome assembly statistics. GC (Guanine and cytosine percentage)

	macOpe2
Number of contigs	162
Assembled genome length	411228032
GC (%)	43.93
N50	19217602
N90	12148816
L50	12
L90	24

Table S2: Summary of the repeats annotation using repeatsmasker.

Repeat class	Repeat family	Sub-family	Number of elements	Total length (bp)	Percentage in the genome (%)
Retroelements			116391	32955420	6.78
	SINEs:		6273	1017016	0.21
		Penelope	9438	2715604	0.56
	LINEs:		61426	16447763	3.38
		L2/CR1/Rex	38220	10460451	2.15
		R1/LOA/Jockey	1434	233297	0.05
		R2/R4/NeSL	2154	544878	0.11
		RTE/Bov-B	7389	1528880	0.31
		L1/CIN4	2176	736663	0.15
	LTR:		48692	15490642	3.19
		BEL/Pao	4151	841670	0.17
		Ty1/Copia	468	174882	0.04
		Gypsy/DIRS1	10226	4243390	0.87
		Retroviral	23537	8392820	1.73
DNA transposons			52062	11076209	2.28
	hobo/activator		15182	4804346	0.99
	Tc1-IS630-Pogo		17345	3178319	0.65
	PiggyBac		12813	18397776	0.38
Rolling-circles			7115	1119332	0.23
Unclassified			15006	2073092	0.43
Satellites			8885	1707277	0.35

Table S3: Summary of the contigs containing telomeric sequences

contig name	telomere start	telomere end	Chromosome ID
ptg000001l	0	7800	
ptg000002l	1400	5600	
ptg000004l	0	6400	Chr3
ptg000004l	20357400	20365586	Chr3
ptg000006l	19384400	19393576	
ptg000007l	600	5400	
ptg000008l	0	7800	
ptg000009l	24015400	24022457	
ptg000010l	0	9000	Chr9
ptg000010l	22105000	22111710	Chr9
ptg000011l	21562200	21566751	
ptg000012l	0	6000	
ptg000015l	0	1000	
ptg000015l	2000	5400	
ptg000016l	0	3000	
ptg000017l	0	3800	
ptg000018l	0	7800	
ptg000019l	0	1000	
ptg000020l	1600	8000	
ptg000024l	0	7400	Chr21
ptg000024l	20760800	20766222	Chr21
ptg000025l	0	1800	
ptg000025l	2200	7400	
ptg000026l	0	5200	Chr17
ptg000026l	17471800	17478600	Chr17
ptg000028l	0	6800	Chr8
ptg000028l	17089800	17096669	Chr8
ptg000030l	0	5600	Chr15
ptg000030l	17874200	17880400	Chr15
ptg000054l	31000	38154	
ptg000083l	0	6800	
ptg000100l	0	7200	
ptg000112l	0	7600	
ptg000120l	24400	26339	

Contigs marked in yellow appear to be fully sequenced telomere-to-telomere

Software	Version	URL	parameters	comment
Trim Galore	0.6.5	https://www.bioinformatics.babraham.ac.uk/projects/trim_galore/	for fastq in *.fastq.gz ; do trim_galore --quality 25 --fastqc --length 25 --output_dir qc_results $fastq ; cd qc_results ; multiqc . ; Done	This line of code was used to trim the adapter sequences from the fastq and to drop the reads having quality value of less than 25. The multiqc was then run in the "qc_results" directory to consolidate the quality control results for ease of interpratation.
Hifiasm	0.19.3	https://github.com/chhylp123/hifiasm	hifiasm -o MacOpe2.asm -t 32 -l 0 hifi_data/*.fastq.gz	The hifiasm was run using default parameters. The number of threads used was 32 (-t 32) and the purging level was set to 0 (-l 0).
BRAKER	3.0.2	https://github.com/Gaius-Augustus/BRAKER	braker.pl --cores=$THREADS --verbosity=3 --makehub --species=$SPECIES_NAME --gff3 --genome=$GENOME --bam=$TRANSCRIPT_BAM --BAMTOOLS_PATH=/data/okendojo/conda/envs/BRAKER/bin/ --softmasking --useexisting --UTR=on	SPECIES_NAME="paradisefish" is the species name to be used when building the Augustus gene model. In --genome=$GENOME is the soft masked reference genome sequence to be analysed ; and --bam=$TRANSCRIPT_BAM is the list of bam files to be used in the analysis.

MAKER	3.01.04	https://github.com/Yandell-Lab/maker	Running MAKER round one: mpiexec -n 32 maker -base maker_001 maker1_opts.ctl maker_bopts.ctl maker_exe.ctl -f ; Running maker round two: mpiexec -n 32 maker -base maker2_blat maker2_opts.ctl maker_bopts.ctl maker_exe.ctl -f	Running MAKER round one ; we specified the MacOpe2.fasta as our genome of interest , the transcriptome assembly from Trinity was specified as esxpressed sequence tag (EST). The zebrafish/vertbrates proteomes was used to do the protein homology evidence analysis. "model organism" was set to simple and the custom repeat librabry generated by RepeatModeler. The genomic repears was softmasked during the first round of MAKER analysis. The second round of MAKER run, the gff file from the first MAKER run was used in the re-annotation. The GeneMark file from the BRAKER run and the augustus was used to run gene predictions.
RepeatModeler	5.8.8	https://www.repeatmasker.org/RepeatModeler/	BuildDatabase -name paradisefish -engine ncbi MacOpe2.fasta ; RepeatModeler -pa	The first run is used to build a new

			32 -engine ncbi -database paradisefish 2>&1 | tee 00_repeatmodeler.log	RepeatModeler BLAST database and the second run does the RepeatModeller analysis of the MacOpe2 genome
RepeatMasker	4.1.5	https://www.repeatmasker.org/	RepeatMasker -pa 32 -species paradisefish -e ncbi -dir macOpe2_mask MacOpe2.fasta	
GenomeScope	1.0.o	https://github.com/schatzlab/genomescope	Rscript genomescope.R MacOpe2.histo 21 150 output_dir	21 is the k-mer length and 150 is the maximum read length.
Jellyfish	2.3.0	https://github.com/gmarcais/Jellyfish	jellyfish count -C -m 21 -s 2000M -t 32 <(zcat L001_R1_001.fastq.gz L001_R2_001.fastq.gz) <(zcat L004_R1_001.fastq.gz L004_R2_001.fastq.gz) -o MacOpe2.jf ; jellyfish histo -t 32 MacOpe2.jf > MacOpe2.histo	Jellyfish was used to count kmers. The second part of the jellyfish run was used to export the k-mer count histogram. The length of mer was set 21 (-m 21) and and the initial harsh size was set at 2000M (-s 2000M).
Trinity	2.0.2	https://github.com/trinityrnaseq/trinityrnaseq.github.io	Trinity --seqType fq --max_memory 180G --samples_file sample.txt --trimmomatic --monitoring --monitor_sec 30 --CPU 32 --output MacOpe2_trinityasm	"sample.txt" contains the fullpaths of fastq files to be used in the assembly.
BUSCO	5.4.6	https://busco.ezlab.org/	busco -i assembly.fasta -o MacOpe2 -m genome --long --augustus_parameters='--progress=true' --augustus_species paradise_fish --auto-lineage-euk -f --cpu 32 --augustus --out_path results	
QUAST	5.2.0	https://github.com/ablab/quast	quast.py -o ${outdir} -l 'MacOpe2_hifiasm, verkko_asm, Haplotype1, Haplotype2' -t 32 --eukaryote --est-ref-size 411228032 --plots-format png MacOpe2.fasta	

			verrko_assembly.fasta hap1.fasta hap2.fasta	
rnaQUAST	2.2.3	https://github.com/ablab/rnaquast	python rnaQUAST.py --transcripts trinity.fasta --reference MacOpe2.fasta --gtf MAKER.gtf	
K-mer analysis toolkit (KAT)	2.4.1	https://github.com/TGAC/KAT	kat comp -t 16 -o pe_vs_assembly 'ERR3332352_?.fastq.gz' MacOpe2.fasta	
GATK4	4.4.0.0	https://github.com/broadinstitute/gatk	Reads mapping to assembled genome: dragen-os -r dragenRef -1 ERR3332352_1.fastq.gz -2 ERR3332352_2.fastq.gz > macOpe2.sam ; Get str table: gatk ComposeSTRTableFile -R MacOpe2.fasta -O str_table.tsv ; Convert sam to bam then sort and index: samtools view -S -b macOpe2.sam -o macOpe2.bam ; samtools sort macOpe2.bam -o macOpe2.sorted.bam ; samtools index macOpe2.sorted.bam ; Add readgroups : java -jar $PICARDJARPATH/picard.jar AddOrReplaceReadGroups -I macOpe2.bam -O macOpe2_RG.bam -RGID 4 -RGLB lib1 -RGPL ILLUMINA -RGPU unit1 -RGSM 20 -SO coordinate --CREATE_INDEX true ; Calibrate model: gatk CalibrateDragstrModel -R MacOpe2.fasta -I macOpe2.sorted.bam -str str_table.tsv -O dragstr_model.txt ; Call variants : gatk HaplotypeCaller -R macOpe2.fasta -I macOpe2.sorted.bam -O sv_output_file.vcf --dragen-mode true --add-output-vcf-command-line false --dragstr-params-path dragstr_model.txt ; gatk VariantFiltration -V output_file.vcf --filter-expression "QUAL < 10.4139" --filter-name "DRAGENHardQUAL" -O output_filtered.vcf	
OrthoFinder	2.5.5	https://github.com/davidemms/OrthoFinder	OrthoFinder -f MacOpe2_results ; The visualization of the data was then done in R	"MacOpe2_results" contains protein sequences from

				MacOpe2 MAKER run, medaka, zebrafish.
